# Geriatric Emergency Management Programs and Days at Home Among People Living With Dementia in Ontario

**DOI:** 10.1111/acem.70437

**Published:** 2026-11-04

**Authors:** Margret Lo, James Lee, Emily Bagley, Humberto Omana, Fabrice Mowbray, Iwona Bielska, Susan Bronskill, Don Melady, Kelly Kay, Lauren Lapointe‐Shaw, Jennifer Watt, Aaron Jones

**Affiliations:** ^1^ Michael G. DeGroote School of Medicine Hamilton Ontario Canada; ^2^ Institute for Clinical Evaluative Sciences Toronto Ontario Canada; ^3^ St Joseph's Health System Hamilton Ontario Canada; ^4^ Regional Geriatric Program of Southwestern Ontario London Ontario Canada; ^5^ St. Joseph's Health Care London London Ontario Canada; ^6^ School of Health Studies Western University London Ontario Canada; ^7^ Lawson Research Institute London Ontario Canada; ^8^ College of Nursing Michigan State University East Lansing Michigan USA; ^9^ Institute of Public Health Jagiellonian University Krakow Poland; ^10^ Department of Health Research Methods, Evidence, and Impact McMaster University Hamilton Ontario Canada; ^11^ Dalla Lana School of Public Health University of Toronto Toronto Ontario Canada; ^12^ Department of Family and Community Medicine, Schwartz‐Reisman Emergency Medicine Institute Mount Sinai Hospital, University of Toronto Toronto Ontario Canada; ^13^ Provincial Geriatrics Leadership Ontario Toronto Ontario Canada; ^14^ Division of General Internal Medicine and Geriatrics Sinai Health System and The University Health Network Toronto Ontario Canada; ^15^ Department of Medicine University of Toronto Toronto Ontario Canada; ^16^ Division of Geriatric Medicine St. Michael's Hospital, Unity Health Toronto Toronto Ontario Canada

## Abstract

**Background:**

People living with dementia frequently present to emergency departments (EDs) and are at increased risk of hospitalization, institutionalization, and other adverse outcomes. Geriatric Emergency Management (GEM) programs are designed to improve emergency care for older adults, but their effectiveness among people living with dementia remains uncertain. We evaluated the association between attendance at an ED with an active GEM program and days alive and at home among older adults living with dementia.

**Methods:**

We conducted a population‐based retrospective cohort of Ontario residents aged 65 years or older with identified dementia who had an index ED visit between April 1, 2021, and March 31, 2024. The exposure was attendance at an ED with an active GEM program.

**Results:**

The cohort included 145,874 individuals, including 110,288 who presented to EDs with GEM programs and 35,586 who presented to EDs without GEM programs. The median age of the population was 81 years and 58.5% of participants were female. After propensity score overlap weighting and adjustment for facility characteristics, individuals who visited GEM EDs spent 7.5 (95% CI, 3.1–12.0) more days alive and at home during the following year than those who visited non‐GEM EDs. Approximately half of this difference was attributable to fewer days spent in long‐term care homes. Visits to GEM EDs were also associated with fewer subsequent ED visits, although index ED length of stay and hospital admission rates were higher.

**Conclusions and Relevance:**

Among older adults living with dementia, care in EDs with GEM programs was associated with more days alive at home over the following year. These findings support continued investment in GEM programs to promote aging at home and to delay institutionalization among people living with dementia.

## 1 Introduction

People living with dementia account for nearly 7% of the emergency department (ED) visits among older adults in North America [1]. Compared with other older adults, people living with dementia visit the ED more frequently and are more likely to experience repeated visits and hospital admissions following ED care [2, 3]. While the ED is an important location of care for older adults, people living with dementia are at increased risk of experiencing ED‐related adverse events, including delirium, falls, dehydration, medication errors, and infections [4]. Repeated visits to the ED with non‐specific symptoms are common among individuals living with dementia and may indicate a lack of appropriate care in the community [2].

The number of people living with dementia in Canada is expected to triple between 2020 and 2050, underscoring the need to implement optimal ED care processes for this population [5]. Geriatric emergency medicine seeks to transform the care of older adults by focusing on the unique physiological, cognitive, and social needs of the population. People living with dementia are a priority group within this field, due to the higher likelihood of atypical presentations, greater clinical complexity, and heightened vulnerability to adverse events [6, 7].

In Canada, geriatric emergency medicine initiatives have included national practice recommendations, specialized clinical training, and nurse‐led geriatric emergency management (GEM) programs [8]. GEM programs provide specialized, frailty‐focused care to older adults with the aim of improving outcomes and patient experiences [9]. In Ontario, GEM programs are staffed primarily by registered nurses with gerontological nursing training and experience, working increasingly in collaboration with other interprofessional team members (e.g., social workers, rehabilitative care professionals, care coordinators, pharmacists) to consult on older adult presentations in the ED. Typical GEM activities include targeted geriatric assessment, individualized care and discharge planning, and coordination with community care and other specialized geriatric services. GEM programs in Ontario started as a pilot project in 1994 with a major expansion in 2004 [10]. GEM programs have expanded periodically in selected locations since then, and while many programs have developed with common characteristics, there is also substantial variation in program availability and structure.

Ontario GEM programs share several clinical features with the American College of Emergency Physicians' Geriatric Emergency Department Accreditation (GEDA) framework, including geriatric expertise, targeted assessment, and coordination of care transitions. GEDA recognizes three levels: Level 3 (bronze), Level 2 (silver), and Level 1 (gold), with progressive requirements. The functions of GEM clinicians overlap with the nurse case management and transitional care roles specified for Levels 1 and 2. However, GEDA accreditation also encompasses departmental leadership, staff education, clinical protocols, quality improvement, outcome monitoring, equipment, and the physical environment. The presence of a GEM program alone therefore does not establish equivalence to any GEDA accreditation level.

While recommendations on best practices for geriatric emergency care have been established in Canada, evidence regarding the impact of these practices on patient outcomes remains limited [11]. Despite a small number of return‐on‐investment evaluations, a province‐wide evaluation of GEM programs in Ontario has not been conducted to date. A better understanding of the relationship between GEM programs and outcomes for people living with dementia could therefore inform guidelines and recommendations that improve the quality of life for this population. The objective of our study was to examine the association between attendance at an ED with a GEM program and subsequent days at home and healthcare use outcomes among people living with dementia in Ontario.

## 2 Methods

### 2.1 Study Design and Setting

We conducted a population‐based retrospective cohort study in Ontario, Canada. Ontario is Canada's most populous province with over 16.1 million people, including three million individuals over the age of 65 [12, 13]. Healthcare in Ontario is administered primarily via a universal publicly funded health insurance system that covers medically necessary care including physician services, hospital care, ED services, and prescription medications for adults aged ≥ 65 years.

### 2.2 Data Sources

We accessed data from several population‐based health administrative databases. The data were linked using unique encoded identifiers and analyzed at ICES (formerly the Institute for Clinical Evaluative Sciences). ICES is an independent, non‐profit research institute whose legal status under Ontario's health information privacy law allows it to collect and analyze healthcare and demographic data, without consent, for health system evaluation and improvement. We accessed physician billing information from the Ontario Health Insurance Plan (OHIP) database, emergency department visits from the National Ambulatory Care Reporting System (NACRS), hospital admissions from the Discharge Abstract Database (DAD), inpatient rehabilitation from the National Rehabilitation Reporting System (NRS), inpatient mental health from the Ontario Mental Health Reporting System (OMHRS), residential care from the Continuing Care Reporting System (CCRS), community‐dispensed medications from the Ontario Drug Benefit (ODB) and basic demographics and health insurance eligibility from the registered persons database (RPDB). Data on the availability of GEM programs were extracted from the Specialized & Focused Geriatric Services Asset Inventory, which is a publicly available dataset that is annually curated by Provincial Geriatrics Leadership Ontario.

### 2.3 Population

We included adults, 65 years and older, with dementia who visited an emergency department in Ontario between April 1, 2021, and March 31, 2024. Visits among residents of long‐term care homes were excluded. Dementia status was ascertained using a validated algorithm that combines diagnostic codes from physician visits and hospital admissions, and dispensations of dementia‐specific medications [14]. If an individual had more than one eligible ED visit during the time period, only the first visit was retained.

### 2.4 Measures

#### 2.4.1 GEM Programs

EDs with active GEM programs were identified from the geriatric services asset inventory and linked by facility name and year to facilities in the administrative data sets. The exposure was attendance at an ED with an active GEM program. We were not able to distinguish programs according to staffing, hours of coverage, assessment processes, or integration with other services.

#### 2.4.2 Outcomes

The primary outcome was days alive and at home in the year after the index ED visit [15]. This outcome combines data from multiple sources to determine how many days an individual spent in hospitals, long‐term care homes, other inpatient facilities, or was deceased in the 365 days after the index ED visit, with the rest of the days assumed to have been alive and at home. Each hospital or other institutional episode that occurs during the year after the index date contributes to this measure. We chose this as the primary outcome as it combines health system use and mortality data into a single measure, avoiding the methodological challenges of variable follow‐up time, censoring, and competing risks that arise from examining individual health system use outcomes such as repeated ED visits. Days at home is also a patient‐centered outcome that aligns with the priority of aging in the community [16]. As a secondary analysis, we measured the days spent at home and in each setting over the 180 days following the index ED visit. We also extracted secondary outcomes on ED use, hospital admissions, and total health system costs in the 30 and 180 days following the index ED visit, and death or long‐term care home placement within a year.

### 2.5 Patient and Facility Characteristics

At the individual level we captured basic demographics and neighborhood‐level measures of socioeconomic status at baseline. Rurality was defined as community size of less than 10,000 as per the 2016 Canadian Census and based on standard Statistics Canada definitions [17]. We reported on the prevalence of asthma, congestive heart failure, diabetes, chronic obstructive pulmonary disease, and hypertension using validated administrative data algorithms [18]. We calculated a frailty index based on the CIHI Hospital Frailty Risk Measure (HFRM) [19]. The HFRM is calculated as the proportion of 36 frailty‐related health deficits identified from prior hospital and emergency department diagnostic codes, yielding a score from 0 to 1, with higher scores indicating greater frailty. We reported whether an individual was prescribed specific medication classes (benzodiazepines, antipsychotics, or drugs with a strong anticholinergic effect based on the Beers criteria [20, 21]) at baseline. We identified the use of publicly funded home care, an ED visit, or a hospital admission in the previous six months. Related to the index ED visit, we determined whether the individual arrived by ambulance, the triage level of the visit (based on the Canadian Triage Acuity Scale (CTAS) score), and most responsible diagnosis code (first three characters of ICD‐10‐CA). We also reported ED length of stay (LOS) and if the patient was admitted, the hospital LOS. For those admitted to hospital from the index visit we reported the proportion designated alternate level of care (ALC), which is an administrative designation that occurs where there is no longer a medical reason for the patient to remain in hospital. At the ED‐facility level, we reported the community size where the ED was located as well as the annual volume of adult ED visits.

### 2.6 Analysis

We presented the characteristics of the individuals and ED facilities included in the study with descriptive statistics, stratified by GEM status of the ED facility. We fit an individual‐level propensity score model using logistic regression including demographics, chronic conditions, health status, and ED visit characteristics (triage level, ambulance arrival). Using the propensity scores, we calculated and applied normalized overlap weights to balance individual characteristics between the GEM and non‐GEM patient groups [22]. Overlap weights provide bounded, stable weights that emphasize the population with near equal chances of belonging in either treatment group. Overlap weights produce exact mean covariate balance when the propensity score is derived with logistic regression [23].

We reported all outcomes in both the unweighted (raw) and overlap‐weighted samples. For our primary outcome of days spent alive and at home, we used weighted linear regression models with empirical standard errors to account for clustering at the level of the ED facility. We included ED rurality as a five‐level categorical variable and annual adult ED volume as covariates. We reported the mean difference in the number of days alive and at home at 365 days and 180 days with 95% confidence intervals. We repeated this analysis for days spent in each setting.

### 2.7 Sensitivity Analysis

We repeated our primary analysis using weighted linear mixed models with random intercepts at the ED facility level and empirical standard errors to test sensitivity to an alternate method of clustering adjustment.

### 2.8 Ethics

We received ethics approval for this study from the Hamilton Integrated Research Ethics Board (17581).

## 3 Results

We included 145,874 individuals living with dementia who visited an ED in Ontario between April 1, 2021 and March 31, 2024. ED visits occurred at 161 different facilities, of which 66 (41.0%) had active GEM programs at the time of visit. Of the total number of ED visits, 110,288 (75.6%) were to GEM facilities. Except for rurality, patient characteristics were similar between the GEM and non‐GEM groups (Table 1). Median age at index ED visit was 81 years in the GEM group and 80 in the non‐GEM group, with the GEM group having a slightly higher proportion of females (58.9% vs. 57.4%). Only 5.0% of individuals who visited GEM facilities resided in rural locations, compared to 29.9% of those who visited non‐GEM facilities. The mean frailty score was similar between the two groups, and individuals in the GEM group were slightly more likely to be living with hypertension, diabetes, asthma, and congestive heart failure. Medication use and prior health system use were largely similar at baseline.

**TABLE 1:** Characteristics of individuals living with dementia in Ontario, Canada visiting an ED between April 1, 2021 and March 31, 2024.

Characteristics, *n* (%)	Patient characteristics	
	Visited a GEM ED *n* = 110,288	Visited a non‐GEM ED *n* = 35,586
Demographics		
Age, yrs., median (Q1, Q3)	81 (75, 86)	80 (75, 86)
Sex, F	64,914 (58.9)	20,425 (57.4)
Rural residence	5493 (5.0)	10,644 (29.9)
Health Status		
Hypertension	93,230 (84.5)	29,140 (81.9)
Diabetes	42,379 (38.4)	12,608 (35.4)
Asthma	19,855 (18.0)	5320 (14.9)
Congestive Heart Failure	24,111 (21.9)	7125 (20.0)
COPD	31,150 (28.2)	11,078 (31.1)
HFRM, mean (SD)	0.13 (0.10)	0.13 (0.10)
Medication use at baseline		
Antipsychotics	16,442 (14.9)	5206 (14.6)
Benzodiazepines	6462 (5.9)	2082 (5.9)
Medications with strong anticholinergic properties	8078 (7.3)	3092 (8.7)
Health system use		
Public home care use in past 6 months	62,983 (57.1)	19,621 (55.1)
ED visit in past 6 months	23,451 (21.3)	8463 (23.8)
Hospital admission in past 6 months	16,820 (15.3)	5330 (15.0)
Index ED visit characteristics		
Arrived by Ambulance	72,912 (66.1)	21,182 (59.5)
CTAS: Triage Scale		
1: Resuscitation	2564 (2.3)	689 (1.9)
2: Emergent	29,872 (27.1)	7908 (22.2)
3: Urgent	69,759 (63.3)	20,617 (57.9)
4: Less Urgent	6651 (6.0)	5000 (14.1)
5: Non‐Urgent	1332 (1.2)	1221 (3.4)

Abbreviations: COPD, Chronic obstructive pulmonary disease; CTAS, Canadian Triage Acuity Level; HFRM, Hospital frailty risk measure.

### 3.1 Index ED Visit Characteristics

Visits to GEM facilities were more likely to be triaged at higher acuity levels. For example, 7.2% of visits to GEM facilities were considered less‐urgent or non‐urgent compared to 17.5% of visits to non‐GEM facilities (Table 1). The most common primary diagnostic codes were broadly similar between the groups, with the top 8 codes being the same, though not in the same order of frequency. The most common diagnosis in both groups was “Other disorders of the urinary system”, which includes urinary tract infections. The top 10 presenting complaints were also highly similar between the groups, with the most frequent complaint being “general weakness” (Supplemental Table 1).

ED facilities without GEM programs were far more likely to be in communities with a size of less than 10,000 (63.2% vs. 12.1%) compared to those with GEM programs, while facilities with GEM programs were more likely to be in the largest community size (33.3% vs. 5.3%). GEM facilities were more common in Toronto and Central Ontario, with facilities without GEM programs more frequent in the North and West regions. The mean annual volume in EDs with GEM programs was more than double that of the non‐GEM programs (Table 2).

**TABLE 2:** ED facility characteristics, Ontario, 2021–2024.

Characteristics	Facility characteristics	
	EDs with a GEM program (*n* = 66)	EDs without a GEM program (*n* = 95)
Annual adult ED volume, (mean)	96,053	41,879
Community size		
1,500,000 or more	22 (33.3%)	5 (5.3%)
500,000–1,499,999	12 (18.2%)	8 (8.4%)
100,000–499,999	10 (15.2%)	9 (9.5%)
10,000–99,999	12 (18.2%)	8 (8.4%)
Less than 10,000	8 (12.1%)	60 (63.2%)
Missing	2 (3.0%)	5 (5.3%)
Ontario Health Region		
Central	15 (22.7%)	6 (6.3%)
East	16 (24.2%)	19 (20.0%)
North East	8 (12.1%)	20 (21.1%)
North West	0 (0.0%)	13 (13.7%)
Toronto	10 (15.2%)	1 (1.1%)
West	17 (25.8%)	36 (37.9%)

### 3.2 Outcomes: Days at Home in the Year Following Index ED Visit

Unweighted results describe the observed outcomes in the original cohort while weighted results apply propensity score overlap weights to generate results in a cohort that is balanced on measured patient and index visit characteristics. After weighting, the GEM and non‐GEM groups were balanced on all measured characteristics (Supplemental Table 2). In the unweighted sample, individuals who visited GEM facilities spent 2.0 (0.3, 3.6) fewer days at home in the year after the index visit. However, after weighting and further adjustment for facility community size and ED volume in regression models, we estimated that individuals who visited GEM facilities spent 7.5 (3.1, 12.0) more days alive and at home in the year following the index ED visit. This means that patients who attended an ED with a GEM program spent, on average, approximately one additional week alive and at home during the year after the visit compared to those who attended an ED without an active GEM program. Roughly half of the difference was accounted for by fewer days spent in long‐term care 3.5 (1.6, 5.5) (Table 3). Results at 180 days were consistent with the one‐year results. We estimated that individuals spent 3.5 (1.0, 5.9) additional days at home within the 180 days following the index visit with 1.0 (0.2, 1.9) fewer days spent in a long‐term care home.

**TABLE 3:** Mean and mean difference in number of days spent in various settings in one year and 180 days following a visit to a GEM ED facility in Ontario, 2021–2024.

Follow‐up window^1^	Unadjusted				Adjusted^2^
	Location	GEM	Non‐GEM	Difference (95% CI)	Difference (95% CI)
1 year	At home	261.8	263.7	−2.0 (−3.6, −0.3)	7.5 (3.1, 12.0)
	Hospital	19.1	18.7	0.3 (−0.1, 0.8)	−1.5 (−4.1, 1.0)
	Long‐term care home	22.8	27.5	−4.7 (−5.5, −3.8)	−3.5 (−5.5, −1.6)
	Deceased	53.0	48.4	4.6 (3.3, 5.9)	−2.6 (−5.8, 0.5)
180 days	Other^3^	9.1	7.6	1.5 (0.3, 2.8)	−0.1 (−2.3, 2.0)
	At home	135.0	137.1	−2.1 (−2.9, −1.3)	3.5 (1.0, 5.9)
	Hospital	13.7	13.3	0.4 (0.1, 0.7)	−1.4 (−3.2, 0.4)
	Long‐term care home	7.9	9.3	−1.4 (−1.8, −1.0)	−1.0 (−1.9, −0.2)
	Deceased	17.7	15.6	2.1 (1.5, 2.6)	−1.0 (−2.3, 0.3)
	Other^3^	5.7	4.7	1.0 (−0.1,2.0)	−0.2 (−1.5, 1.1)

^1^ Outcome includes days spent during all encounters in the window.

^2^ From linear regression model featuring overlap weights for individual and index ED visit characteristics and adjustment for with ED community size and volume.

^3^ Includes inpatient rehabilitation, complex continuing care hospital, inpatient mental health, and emergency departments.

### 3.3 Outcomes: Health System Use

Unweighted and weighted secondary outcomes are presented in Tables 4 and 5, respectively. In the weighted comparison, individuals attending GEM EDs had longer mean stays during the index ED visit (12.7 vs. 9.6 h) and were more likely to be admitted to hospital from that visit (36.2% vs. 34.1%). Among those admitted, mean hospital length of stay was shorter in the GEM group (16.2 vs. 17.8 days). The median length of stay, however, was the same in both groups at 8 days. During the subsequent 180 days, individuals attending GEM EDs were less likely to have another ED visit (46.7% vs. 50.6%; an absolute difference of 3.9 percentage points), although hospital admission was slightly more frequent (39.1% vs. 38.0%). Admission to long‐term care within 1 year was less frequent in the GEM group (17.6% vs. 21.9%). Mean health system costs over 180 days were higher ($32,032 vs. $29,877 CAD), while one‐year mortality was 21.6% in the GEM group and 22.1% in the non‐GEM group.

**TABLE 4:** ED and health system use outcomes following index ED visit, unweighted sample.

Outcomes	Unweighted sample		
	GEM	Non‐GEM	Difference (95% CI)
Index visit			
ED LOS (hours), mean	13.4	9.4	4.0 (3.8, 4.2)
Admitted to hospital from index	38.9%	32.8%	6.2% (5.7%, 6.8%)
Hospital length of stay if admitted (days), mean^1^	16.0	18.0	−2.1 (−2.7, −1.5)
Hospital length of stay if admitted (days), median^1^	8.0	8.0	0.0 (−0.25, 0.25)
ALC stay if admitted	29.4%	28.4%	1.0% (0.1%, 1.9%)
After index visit			
ED visit within 30 days	18.8%	21.9%	−3.1% (−3.6%, −2.7%)
ED visit within 180 days	46.8%	50.8%	−4.0% (−4.6%, −3.4%)
Hospital admission within 30 days	24.9%	21.2%	3.8% (3.3%, 4.3%)
Hospital admission within 180 days	40.4%	37.3%	3.1% (2.5%, 3.7%)
Mean health system cost within 180 days (CAD)	$33,391	$29,323	$4068 ($3715, $4603)
Admitted to a long‐term care home within one year	18.0%	21.7%	−3.7% (−4.2%, −3.3%)
Died within a year	22.8%	21.6%	1.2% (0.7%, 1.6%)

^1^ Hospital length of stay includes only the index visit.

**TABLE 5:** ED and health system use outcomes following index ED visit, weighted sample.

Outcomes	Weighted sample		
	GEM	Non‐GEM	Difference (95% CI)
Index visit			
ED LOS (hours), mean	12.7	9.6	3.05 (2.9, 3.2)
Admitted to hospital from index	36.2%	34.1%	2.1% (1.5%, 2.7%)
Hospital length of stay if admitted (days), mean^1^	16.2	17.8	−1.6 (−2.2, −1.0)
Hospital length of stay if admitted (days), median^1^	8.0	8.0	0.0 (−0.23, 0.23)
After index visit			
ED visit within 30 days	18.9%	21.8%	−2.9% (−3.4%, −2.4%)
ED visit within 180 days	46.7%	50.6%	−3.9% (−4.5%, −3.3%)
Hospital admission within 30 days	23.8%	21.7%	2.1% (1.6%, 2.6%)
Hospital admission within 180 days	39.1%	38.0%	1.2% (0.6%, 1.8%)
Mean health system cost within 180 days (CAD)	$32,032	$29,877	$2155 ($1714, $2597)
Admitted to a long‐term care home within one year	17.6%	21.9%	−4.4% (−4.8%, −3.95%)
Died within a year	21.6%	22.1%	−0.5% (−1.0%, 0.0%)

^1^ Hospital length of stay includes only the index visit.

### 3.4 Sensitivity Analysis

Using the linear mixed models, the average number of additional days spent alive and at home in the GEM group was attenuated to 4.5 (−0.6, 9.6). The average number of long‐term care home days avoided was similar at 3.2 (1.1, 5.2) (Supplementary Table 3).

## 4 Discussion

We used linked, population‐based health system data to examine outcomes related to attendance at EDs with and without GEM programs in Ontario. After adjustment for confounding, we found that individuals living with dementia who visited EDs with a GEM program spent an additional 7.5 days alive and at home in the year following the visit compared to those who visited an ED without a GEM program. Approximately half of this difference was due to fewer days spent in a long‐term care home. Attendance at GEM facilities was also associated with fewer subsequent ED visits, but longer ED stays and a greater likelihood of hospital admission during the index encounter.

Our findings add to the emerging international evidence base on geriatric emergency care models. While the published literature broadly supports the effectiveness of geriatric emergency care, there is significant heterogeneity in outcomes with few multi‐site studies conducted [24, 25]. Prior studies of Ontario's GEM programs have been limited in scope but generally concluded that the programs improved patient and system outcomes [26, 27, 28]. Our study extends this literature by studying GEM programs across Ontario, focusing specifically on individuals living with dementia, a population at a particularly high risk of adverse outcomes following ED presentation. Our use of days alive and at home as the primary outcome is an important novel element, as it is a patient‐oriented outcome that can summarize the complexity of post‐ED pathways [29, 30].

Our finding that attendance at a GEM ED was associated with more days spent at home is highly relevant to health systems seeking to preserve independence and reduce avoidable institutional care among growing populations of older adults with complex needs [31]. Although we found that GEM programs were associated with longer ED lengths of stay, this may reflect more comprehensive assessments and detailed discharge planning involved in geriatric care. Current Ontario GEM staffing models vary by hours of coverage, which can result in patients being held in ED until they can be seen by GEM. Higher index admission rates in GEM facilities but lower subsequent ED visits may also suggest that GEM provides improved recognition of patients requiring medical management and stabilization. Despite spending more time in EDs, patients who were admitted at EDs with GEM programs had significantly shorter mean lengths of stay with similar median lengths of stay. This suggests possible improvement in removing barriers to discharge that may otherwise result in extended stays. Together these findings indicate that GEM may provide greater intensity of care during the index encounter, while also being associated with improved downstream outcomes over the months following the visit.

We found that the majority of EDs in Ontario did not have GEM programs, and the existing programs were clustered in urban and centralized locations. Given the positive findings of our study, this uneven distribution of specialized geriatric care may contribute to inequity in aging outcomes. Healthcare systems should consider continued expansion and funding of GEM programs within EDs, with a particular focus on increasing access in rural and less centralized areas. However, rural expansion will need to consider local healthcare infrastructure and availability of community resources, as much of the effectiveness of the program may rely on connections with community‐based supports. Future research that evaluates specific components of GEM programs and the mechanisms that drive improved outcomes would be valuable in the development of models for regions with fewer resources. While our findings support the effectiveness of GEM, improving aging outcomes for older adults will require more than specialized services in the ED. GEM should be viewed as only one component of a broader, integrated geriatric care strategy spanning emergency, hospital, primary, community, and long‐term care settings.

## 5 Limitations

There are several notable limitations associated with this study. Our health administrative databases did not contain detailed information on cognitive performance, functional status, or social determinants of health of individuals. Data on GEM programs were available only at the facility level rather than the individual patient level; therefore, we were unable to determine which patients received care from a GEM clinician. GEM programs are not standardized and vary in staffing, hours of coverage, assessment processes, and integration with other services. We could not fully characterize this variation or identify which program components were associated with outcomes. GEM programs do not directly align with GEDA accreditation levels and few EDs in Canada have sought accreditation. Finally, our study took place within a single Canadian province and there was limited availability of GEM programs in rural areas, both of which may affect generalizability. However, GEM is an international model and the challenges faced by older adults in the ED are shared across jurisdictions.

## 6 Conclusions

Among people living with dementia, attendance at an ED with a GEM program was associated with more days alive and at home during the following year, including fewer days in long‐term care. Continued investment in GEM programs, particularly in underserved and rural regions, may help health systems better meet the needs of an aging population with increasingly complex care needs.

## Author Contributions

M.L. contributed to study conception and design, data collection, and drafting and revising the manuscript. J.L. contributed to data analysis and interpretation and critical revision of the manuscript. E.B., H.O., F.M., I.B., S.B., D.M., K.K., L.L.‐S., and J.W. contributed to study design and provided critical review and revision of the manuscript. A.J. supervised the project and contributed to study conception and design, methodology, data analysis and interpretation, and drafting and critical revision of the manuscript. All authors reviewed and approved the final manuscript.

## Funding

This study was received funding from the Alzheimer Society of Canada (0000000049). AJ is supported by the Schlegel Chair in Clinical Epidemiology and Aging. JW is supported by the Entente Education Canada Chair in Geriatric Medicine at the University of Toronto.

## Conflicts of Interest

The authors declare no conflicts of interest.

## Supporting information


**Supplemental Table 1:** Top 10 most responsible diagnosis codes (ICD‐10‐CA) and presenting complaints for index ED visits.
**Supplemental Table 2:** Weighted distribution of characteristics following application of normalized overlap weights.
**Supplemental Table 3:** Mean and mean difference in number of days spent in various settings in one year following a visit to a GEM ED facility in Ontario, 2021–2024, sensitivity analysis using linear mixed models.

## Data Availability

The dataset from this study is held securely in coded form at ICES. While legal data sharing agreements between ICES and data providers (e.g., healthcare organizations and government) prohibit ICES from making the dataset publicly available, access may be granted to those who meet pre‐specified criteria for confidential access, available at www.ices.on.ca/DAS (email: das@ices.on.ca). The full dataset creation plan and underlying analytic code are available from the authors upon request.
