# Supplementary material for: Geriatric Emergency Management Programs and Days at Home Among People Living With Dementia in Ontario
Source: Acad Emerg Med. 2026 Nov 4;33(11):e70437. doi: 10.1111/acem.70437 (PMC13736137; doi:10.1111/acem.70437)
Supplement: Supplementary file 1 [file ACEM-33-0-s001.docx]

**Supplemental Table 1:** Top 10 most responsible diagnosis codes (ICD-10-CA) and presenting complaints for index ED visits

| **GEM** |  | **Non-GEM** |  |
| --- | --- | --- | --- |
| **Most responsible diagnosis code (ICD-10-CA)** | **%** | **Most responsible diagnosis code  (ICD-10-CA)** | **%** |
| N39: Other disorders of urinary system | 4.5 | N39: Other disorders of urinary system | 5.3 |
| R53: Malaise and fatigue | 3.2 | F03: Unspecified dementia | 3.3 |
| F05: Delirium due to physiological condition | 2.8 | R53: Malaise and fatigue | 2.9 |
| S09: Other and unspecified injuries of the head | 2.8 | R07: Pain in the throat and chest | 2.6 |
| R07: Pain in the throat and chest | 2.7 | S09: Other and unspecified injuries of the head | 2.4 |
| F03: Unspecified dementia | 2.6 | S01: Open wound of head | 2.4 |
| R41: Other symptoms and signs involving cognitive functions and awareness | 2.5 | R41: Other symptoms and signs involving cognitive functions and awareness | 2.4 |
| S01: Open wound of head | 2.5 | F05: Delirium due to physiological condition | 2.3 |
| U07: Emergency use of | 2.5 | R55: Syncope and collapse | 2.3 |
| S72: Fracture of femur | 2.5 | J18: Pneumonia, unspecified organism | 2.1 |
| **Presenting Complaint** | **%** | **Presenting Complaint** | **%** |
| 7: General weakness | 12.0 | 7: General weakness | 11.7 |
| 407: Head injury | 7.2 | 407: Head injury | 6.2 |
| 651: Shortness of breath | 6.2 | 402: Confusion | 5.5 |
| 402: Confusion | 5.0 | 651: Shortness of breath | 5.0 |
| 557: Lower extremity injury | 4.9 | 557: Lower extremity injury | 4.6 |
| 251: Abdominal pain | 4.5 | 251: Abdominal pain | 4.1 |
| 555: Lower extremity pain | 3.7 | 555: Lower extremity pain | 3.9 |
| 3: Chest pain (cardiac features) | 3.6 | 3: Chest pain (cardiac features) | 3.6 |
| 401: Altered level of consciousness | 3.3 | 556: Upper extremity injury | 3.2 |
| 8: syncope/presyncope | 3.2 | 551: Back pain | 3.0 |

**Supplemental Table 2:** Weighted distribution of characteristics following application of normalized overlap weights

| **Characteristic** | **GEM (%)** | **Non-GEM (%)** | **Standardized Difference** |
| --- | --- | --- | --- |
| Sex, Female | 57.77% | 57.77% | 0.00 |
| Age, 66-70 Years | 10.44% | 10.44% | 0.00 |
| Age, 71-75 Years | 17.35% | 17.35% | 0.00 |
| Age, 76-80 Years | 22.42% | 22.42% | 0.00 |
| Age, 81-85 Years | 23.16% | 23.16% | 0.00 |
| Age, 86+ Years | 26.63% | 26.63% | 0.00 |
| Arrival to ED by Ambulance | 61.74% | 61.74% | 0.00 |
| Asthma | 15.67% | 15.67% | 0.00 |
| Congestive Heart Failure | 20.52% | 20.52% | 0.00 |
| COPD | 30.42% | 30.42% | 0.00 |
| Hypertension | 82.70% | 82.70% | 0.00 |
| Inflammatory Bowel Disease | 1.09% | 1.09% | 0.00 |
| Diabetes | 36.24% | 36.24% | 0.00 |
| Rheumatoid Arthritis | 3.98% | 3.98% | 0.00 |
| Frailty Index, mean | 0.13 | 0.13 | 0.00 |
| CTAS: Resuscitation | 2.06% | 2.06% | 0.00 |
| CTAS: Emergent | 23.80% | 23.80% | 0.00 |
| CTAS: Urgent | 60.62% | 60.62% | 0.00 |
| CTAS: Less Urgent | 10.85% | 10.85% | 0.00 |
| CTAS: Non-Urgent | 2.43% | 2.43% | 0.00 |
| CTAS: Unknown | 0.24% | 0.24% | 0.00 |

**Supplemental Table 3.** Mean and mean difference in number of days spent in various settings in one year following a visit to a GEM ED facility in Ontario, 2021-2024, sensitivity analysis using linear mixed models

|  | **Unadjusted** | | | **Adjusted^2^** |  |
| --- | --- | --- | --- | --- | --- |
|  |  |  |  |  |  |
| **Location^1^** | **GEM** | **Non-GEM** | **Difference** | **Difference (95% CI)** |  |
| At home | 261.8 | 263.7 | -1.9 | 4.5 (-0.56, 9.6) |  |
| Hospital | 19.1 | 18.7 | 0.4 | -0.3 (-3.2, 2.5) |  |
| Long-term care home | 22.8 | 27.5 | -4.7 | -3.2 (-5.2, -1.1) |  |
| Dead | 53 | 48.4 | 4.6 | -1.9 (-4.8, 1.0) |  |
| Other^3^ | 9.1 | 7.6 | 1.5 | -0.8 (-5.0, 3.4) |  |

1. Outcome includes days spent during all encounters in the window
2. From linear regression model featuring overlap weights for individual and index ED visit characteristics and adjustment for with ED community size and volume
3. Includes inpatient rehabilitation, complex continuing care hospital, inpatient mental health, and emergency departments
